# Oxidation modulates LINGO2-induced inactivation of large conductance, Ca^2+^-activated potassium channels

**DOI:** 10.1016/j.jbc.2023.102975

**Published:** 2023-02-02

**Authors:** Srikanth Dudem, Pei Xin Boon, Nicholas Mullins, Heather McClafferty, Michael J. Shipston, Richard D.A. Wilkinson, Ian Lobb, Gerard P. Sergeant, Keith D. Thornbury, Irina G. Tikhonova, Mark A. Hollywood

**Affiliations:** 1 Smooth Muscle Research Centre, Dundalk Institute of Technology, Louth, Ireland; 2 Centre for Discovery Brain Sciences, University of Edinburgh, Scotland, United Kingdom; 3 Almac Discovery Ltd, Health Sciences Building, Belfast, NIR, United Kingdom; 4 School of Pharmacy, Queen’s University of Belfast, NIR, United Kingdom

**Keywords:** potassium channel, LINGO subunits, oxidation-reduction, biophysics, electrophysiology, leucine rich repeat containing proteins, BK, voltage-activated potassium, cDNA, complementary DNA, Ch-T, Chloramine T, CI, Confidence Intervals, DMF, drug master file, Fmoc, Fluorenylmethyloxycarbonyl, HEK, human embryonic kidney, HRMS, high-resolution mass spectrometry

## Abstract

Ca^2+^ and voltage-activated K^+^ (BK) channels are ubiquitous ion channels that can be modulated by accessory proteins, including β, γ, and LINGO1 BK subunits. In this study, we utilized a combination of site-directed mutagenesis, patch clamp electrophysiology, and molecular modeling to investigate if the biophysical properties of BK currents were affected by coexpression of LINGO2 and to examine how they are regulated by oxidation. We demonstrate that LINGO2 is a regulator of BK channels, since its coexpression with BK channels yields rapid inactivating currents, the activation of which is shifted ∼−30 mV compared to that of BKα currents. Furthermore, we show the oxidation of BK:LINGO2 currents (by exposure to epifluorescence illumination or chloramine-T) abolished inactivation. The effect of illumination depended on the presence of GFP, suggesting that it released free radicals which oxidized cysteine or methionine residues. In addition, the oxidation effects were resistant to treatment with the cysteine-specific reducing agent DTT, suggesting that methionine rather than cysteine residues may be involved. Our data with synthetic LINGO2 tail peptides further demonstrate that the rate of inactivation was slowed when residues M603 or M605 were oxidized, and practically abolished when both were oxidized. Taken together, these data demonstrate that both methionine residues in the LINGO2 tail mediate the effect of oxidation on BK:LINGO2 channels. Our molecular modeling suggests that methionine oxidation reduces the lipophilicity of the tail, thus preventing it from occluding the pore of the BK channel.

Large conductance (G), Ca^2+^, and voltage-activated potassium channels (BK, MaxiK, or KCa1.1) are allosterically activated by an increase in intracellular calcium and depolarization (1, 2). These channels play crucial roles in different physiological functions such as neuronal excitability, neurotransmitter release, and smooth muscle contraction (3, 4). The pore of the BK channel is formed by the tetrameric assembly of α subunits, and its structure has recently been solved using cryo-EM (5, 6). The differing physiological roles of BK channels can be mediated through their association with the regulatory β_1-4_, γ_1-4_, and LINGO1 subunits. Structurally, the β-subunits have two transmembrane helices which are connected extracellularly *via* a large loop and have short intracellular N & C termini tails (7, 8). In contrast, the γ-subunits have a single transmembrane helix with a large extracellular N terminal domain and an intracellular C terminal tail (9, 10, 11). Functionally, the β subunits alter the Ca^2+^ sensitivity of the BK channels to different extents (12, 13), whereas coexpression of either β_2_ or most β_3_ splice variants also induces the inactivation of BKα subunits (14, 15, 16). The γ-subunits shift the voltage-dependent activation of BK channels to negative potentials by varying amounts in the absence of Ca^2+^. Thus, the γ_1_ subunit shifted activation V_1/2_ by ∼-140 mV, whereas γ_2_, γ_3_, and γ_4_ shifted the V_1/2_ by −100 mV, −50 mV, and −20 mV respectively (10).

Recently, LINGO1 has been shown to be a new auxiliary subunit of BK channels, which reduced BK channel plasmalemmal expression, shifted the activation V_1/2_ ∼-60 mV and induced rapid inactivation of the BK channels (11). LINGO1, like the γ_1-4_ subunits, is a leucine-rich repeat-containing protein with a single transmembrane helix and a short intracellular tail. However, in contrast to the γ subunits, LINGO1 contains 12 rather than six LRR domains and possesses an IgI1 domain that is absent in the γ subunits (17, 18). Furthermore, LINGO1 has three homologues named LINGO2, LINGO3, and LINGO4 which share 61%, 56%, and 44% sequence similarity with LINGO-1, respectively (19, 20). However, no study has investigated if any of these homologues also modulate BK channels. In this study, we examined the effects of coexpressing BK and LINGO2 in human embryonic kidney (HEK) cells. The data presented demonstrate that LINGO2 induces the inactivation of BK channels, and this effect can be abolished by oxidation of the LINGO2 subunit.

## Results

**Figure 1:**
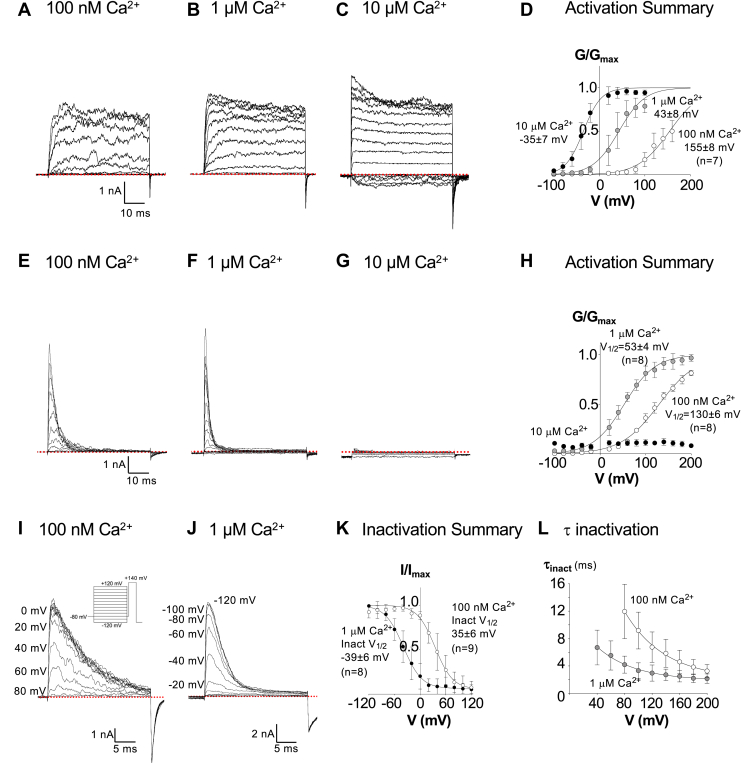
**Biophysical characterization of BKα and BK:LINGO2 currents.** Panels *A*–*C* show typical currents recorded from HEK cell patches expressing BKα subunits. Currents were elicited from −100 mV to +200 mV in 20 mV increments from a −60 mV holding potential. Patches were pre-pulsed to −100 mV before evoking currents, and tail currents were elicited by repolarizing back to −80 mV. The *red dashed lines* in this and other current traces represent zero current. Panel *D* shows a summary, GV curve constructed and fitted with the Boltzmann equation (*solid line*, n = 7). With increased Ca^2+^ concentrations, summary activation V_1/2_ was shifted towards more negative potentials (100 nM, 1 μM, and 10 μM Ca^2+^ - *white, grey,* and *black circles*, respectively). Panels *E*–*G* show that coexpression of LINGO2 with BKα generated rapidly inactivated currents in 100 nM and 1 μM Ca^2+^ concentrations, but currents were practically abolished in 10 μM Ca^2+^. The same voltage clamp protocol was used to record currents as described for *A*–*C*. Panel *H* shows that the activation V_1/2_ in 100 nM [Ca^2+^]_i_ was significantly negatively shifted compared BKα alone (*p* < 0.0001, extra sum of squares F-test). Panels *I* and *J* show the currents evoked by a test step to +140 mV in 100 nM and 1 μM Ca^2+^, respectively, following 200 ms conditioning potentials from −120 mV to +120 mV for 200 ms. This double pulse protocol allowed us to determine the steady state voltage dependence of inactivation, and the voltages shown in *I* and *J* refer to the conditioning potential step. Panel *H* shows a summary I/I_max_ curve plotted from test pulse current amplitudes against the conditioning pulse voltages and fitted with a Boltzmann equation, where the inactivation V_1/2_ was shifted by ∼−75 mV (*p* < 0.0001, extra sum of squares F-test) when the [Ca^2+^] at the cytosolic surface was increased from 100 nM (*open symbols*) to 1 μM Ca^2+^ (*closed symbols*). Panel *I* shows the time constants of inactivation (τ_inact_) in 100 nM (*open symbols*) and 1 μM Ca^2+^ (*filled symbols*). Inactivation at voltages from +40 mV to +200 mV was fitted with single a exponential. Panel *K* shows summary steady state voltage-dependent inactivation curves in 100 nM (*open symbols*) and 1 μM Ca^2+^ (*filled symbols*), whereas Panel *L* shows the inactivation time constants at different voltages in the presence of 100 nM (*open symbols*) and 1 μM Ca^2+^ (*filled symbols*) summary time-dependent changes. BK, voltage-activated potassium; HEK, human embryonic kidney.

Currents were recorded from excised inside out patches of membrane from HEK cells transfected with BKα complementary DNA (cDNA). As shown in Figure 1, *A*–*C* depolarizations from −100 mV to +200 mV in 20 mV increments from a holding potential of −100 mV evoked noisy, sustained currents. These activated at more negative potentials as the [Ca^2+^] at the cytosolic surface of each patch was increased. The effect of Ca^2+^ on the half maximal voltage of activation (V_1/2_) is summarized in the activation curves plotted in Figure 1*D* (n = 7). However, when BKα and LINGO2 cDNA were cotransfected and the cytosolic surface of patches was bathed with 100 nM Ca^2+^ (Fig. 1*E*), the currents activated rapidly at potentials positive to +60 mV and inactivated completely over the course of several milliseconds. In eight experiments summarized in Figure 1*H* (open symbols), the mean V_1/2_ in 100 nM Ca^2+^ was +130 ± 6 mV, compared to (+155 ± 8 mV) recorded in patches expressing only BKα subunits (n = 7, *p* < 0.0001). When [Ca^2+^] was increased to 1 μM (Fig. 1*F*), the currents activated at more negative potentials (V_1/2_=53 ± 4 mV, Fig. 1*H*, grey circles, n = 8), and inactivated more rapidly (grey symbols, Fig. 1*L*), compared to the currents recorded in 100 nM Ca^2+^ (open symbols, Fig. 1*L*). In the presence of 10 μM Ca^2+^ (Fig. 1*G*), only small inward currents were recorded at negative potentials, and outward currents were practically abolished, as demonstrated previously with BK:LINGO1 currents (11). The recovery from inactivation was rapid when patches were stepped to −120 mV for increasing durations, where in six experiments the τ_recovery_ was 0.83 ± 0.28 ms (Fig. S1).

We attempted to assess plasmalemmal expression of BK:LINGO2 channels by measuring BK current amplitude in a series of patches obtained from 5 MΩ pipettes and comparing these with currents recorded under identical recording conditions (100 nM Ca^2+^) from cells transfected with either BKα alone or in combination with LINGO1 cDNA. As Fig. S2*A* suggests, patches from cells transfected with BKα cDNA and depolarized to +160 mV in 100 nM Ca^2+^ produced large, noisy, sustained currents. In contrast, although both BK:LINGO1 (Fig. S2*B*)- and BK:LINGO2 (Fig. S2*C*)-containing patches showed rapid inactivation, as previously shown, the amplitude of the BK:LINGO1 currents was much smaller (11) than that recorded from patches obtained from cells transfected with BKα alone or BK and LINGO2 cDNA. This was reflected in the summary data shown in Fig. S2*D* where the mean peak current in BKα patches (3313 ± 3260 pA, n = 43) was significantly larger than that recorded from BK:LINGO1 patches (166 ± 215 pA, n = 29), but not significantly different from that recorded from BK:LINGO2 patches (2081 ± 2605 pA, n = 43, ANOVA).

When Flag-tagged BKα proteins were coimmunoprecipitated from HEK cell lysates, the HA-tagged LINGO2 protein of the expected size was immunoblotted in three separate experiments (Fig. S3), suggesting that both proteins closely associate when coexpressed in HEK cells.

To characterize the steady-state voltage dependence of the inactivation in BK:LINGO2 currents, we applied a double pulse protocol (shown inset in Fig. 1*I*), where the patches were subjected to a series of conditioning pre-pulses (from −120 mV to +120 mV in 20 mV increments for 200 ms) prior to being depolarized to a test potential of +140 mV for 25 ms. The peak amplitude of these currents was unaltered when conditioning potentials negative to 0 mV were applied in 100 nM Ca^2+^ (Fig. 1*I*). However, with more positive conditioning potentials, the amplitude of the current evoked by a test step to +140 mV began to decline. For example, when a conditioning potential of +40 mV was applied, peak current amplitude was reduced by ∼50% and was abolished when conditioning potentials were more positive than +80 mV. The rate of inactivation increased and its shifted to more negative potentials when [Ca^2+^] was elevated to 1 μM, as shown in Figure 1*J*. In the experiments summarized in Figure 1*K* the V_1/2_ of inactivation was 35 ± 6 mV (n = 9) and −39 ± 6 mV (n = 8), in the presence of 100 nM Ca^2+^ (open symbols) and 1 μM Ca^2+^ (black symbols), respectively (*p* < 0.0001).

**Figure 2:**
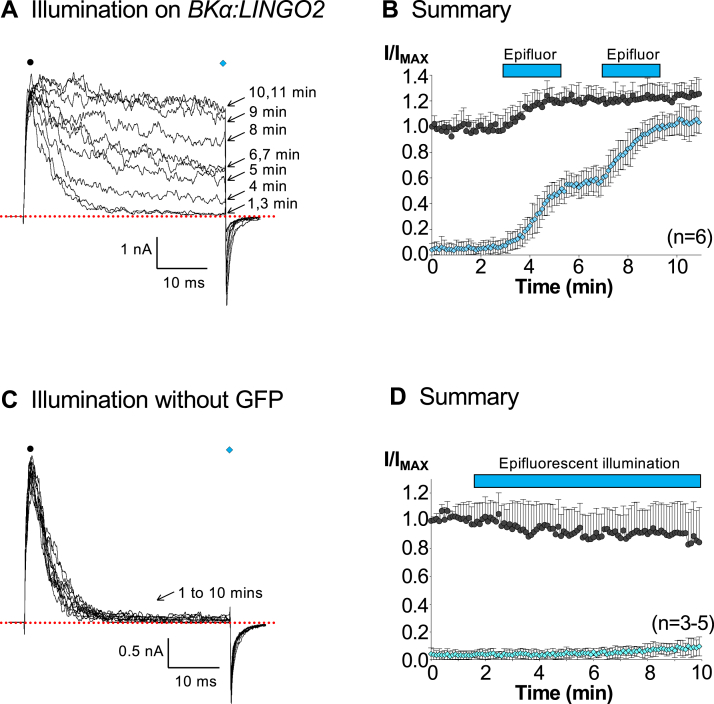
**Epifluorescent illumination abolishes the inactivation of BK:LINGO2 currents and is dependent on GFP.***A*, BK:LINGO2 currents were elicited by stepping to +160 mV from −100 mV, and tail currents were generated by repolarizing back to −80 mV. The *black* and *blue symbols* denote where the peak and sustained current were measured, respectively, to produce the plot shown in Panels *B* and *D*. Panel *B* shows the summary of six experiments where normalized peak (*black symbols*) and sustained (*blue symbols*) current amplitudes were measured during the first and last 5 ms of the depolarization and compared using the Wilcoxon signed-rank test. Patches were illuminated with epifluorescence twice, consecutively for 2 min as denoted by the *blue bars*. At the end of the 4 min of epifluorescence illumination, inactivation was completely abolished in BK:LINGO2 currents (*p* < 0.05 compared to control, Wilcoxon signed-rank test). Panel *C* shows currents evoked (using the same protocol as Panel *A*) from a patch taken from cells in which the GFP plasmid was excluded. Note that illumination failed to remove inactivation when GFP was absent, even when applied continuously for up to 8 min (*p* = 0.06, Wilcoxon signed-rank test). Panel *D* shows summary data for this set of experiments in which the normalized peak (*black circles*) and sustained (*blue diamonds*) current amplitudes were plotted (n = 4–5), where it is clear that the effects of illumination on inactivation were practically abolished. BK, voltage-activated potassium.

Interestingly, we noted that the inactivation observed in the continued presence of 100 nM Ca^2+^ appeared to wane over the course of several minutes in some patches. We investigated this further and found that it was caused by the epifluorescent illumination being left on during some recordings. Figure 2*A* shows typical currents recorded from an excised patch before and during two periods of illumination, applied for 2 min, as denoted by the blue bars in Figure 2*B*. The experimental protocol consisted of a 3 min control period (minutes 1–3) before the first period of illumination was applied (minutes 4 and 5). The second period of illumination was applied during minutes 8 and 9 Figure 2*B* shows summary data for peak (black symbols) and sustained (blue symbols) current amplitude, measured during the first and last 5 ms of the depolarization to +160 mV, respectively. In the absence of illumination, there was no evidence of inactivation waning. However, when the epifluorescence was switched on for the two periods denoted by the blue bars in Figure 2*B*, it was clear that illumination caused a rapid and irreversible removal of inactivation by the end of the second period of illumination. For example, before illumination, the sustained current was 7 ± 5% of the peak current (measured in the first 5 milliseconds of the depolarization) and this increased to 97 ± 10% after a total of 4 min of illumination, (*p* < 0.05, n = 6). The peak current was also increased significantly (by 23 ± 10%, *p* < 0.05). To test if the effects on LINGO2 were dependent on the presence of GFP, we repeated the illumination experiments in patches from cells in which the GFP plasmid was omitted at transfection (Fig. 2, *C* and *D*). Under these conditions, epifluorescent illumination for 4 min failed to significantly increase the sustained current (3 ± 2% *versus* 5 ± 4%) in 5 cells. Although the sustained current increased to 10 ± 7% after a total of 8 min illumination, this effect failed to reach statistical significance (*p* = 0.06). These data suggested that removal of GFP practically abolished the rundown caused by epifluorescent illumination.

**Figure 3:**
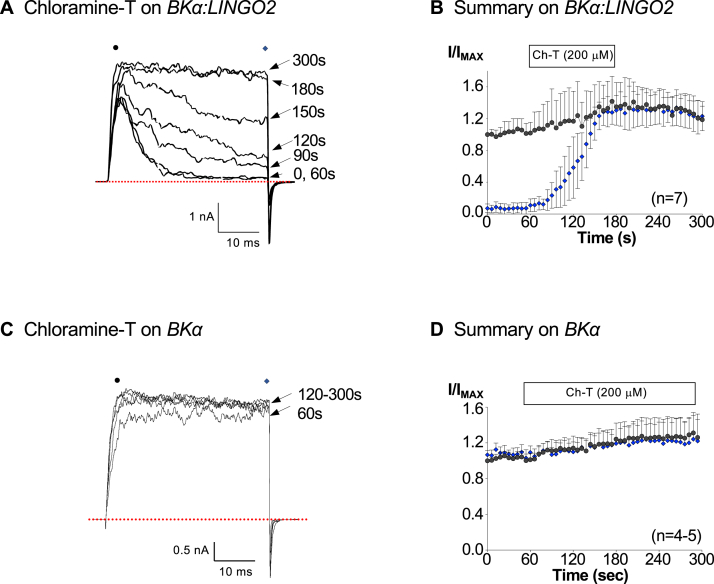
**The oxidizing agent chloramine-T (Ch-T) completely abolished BK:LINGO2 inactivation.** Panel *A* shows the effect of a 2-min application of 200 μM Ch-T on BK:LINGO2 currents elicited by stepping to +160 mV in 100 nM Ca^2+^. These patches were held at −60 mV, and the tail currents were evoked at −80 mV. The sustained current amplitude measured in the last 5 ms of the depolarization was rapidly increased in the continued presence of Ch-T. Panel *B* summarizes seven similar experiments, and both the sustained (*blue symbols*) and peak (*black symbols*) BK:LINGO2 current amplitudes are plotted before, during and after Ch-T application, as denoted by the horizontal bar. Ch-T completely abolished inactivation and also increased peak current amplitude (*p* < 0.05, Wilcoxin signed-rank test). Panel *C* shows the effect of Ch-T on BKα currents using the same voltage protocol as that in Panel *A* and illustrates that it slightly increased their amplitude. Panel *D* shows a summary of 4 to 5 experiments where a 4-min application of Ch-T failed to significantly increase the BKα current amplitude (*p* = 0.43; Wilcoxin signed-rank test). BK, voltage-activated potassium.

We reasoned that illumination could lead to the production of free radicals which may oxidize residues in either the BK channels or the LINGO2 subunits. Consequently, we next tested if the effects of illumination were mimicked by the oxidizing agent, Chloramine T (Ch-T, 200 μM). As Figure 3, *A* and *B* suggest, Ch-T caused rapid abolition of inactivation, such that the sustained current amplitude increased from 11 ± 8% of the peak current to 59 ± 39% (*p* < 0.05) and 130 ± 19% (*p* < 0.05) after 60 s and 120 s of its application, respectively. The amplitude of the peak current was also significantly increased by 35 ± 31% compared to control after 120 s in the presence of Ch-T (*p* < 0.05, Wilcoxin signed-rank test). The effects of Ch-T on peak and sustained current were maintained for the duration of the recording, despite the removal of the oxidizing agent. When we examined the effect of Ch-T on BK currents in the absence of LINGO2, we found that its application for up to 210 s failed to significantly increase current amplitude (21 ± 27%) as shown in Figure 3*D* (n = 4–5, *p* = 0.43).

**Figure 4:**
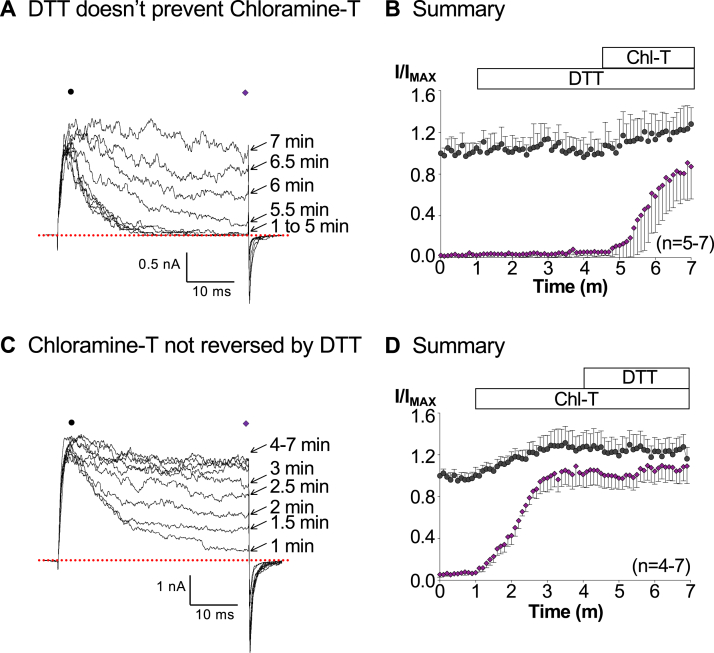
**Effects of Ch-T on BK:LINGO2 currents were insensitive to the reducing agent DTT.** Panel *A* shows a typical current elicited by stepping to +160 mV in 100 nM Ca2+, before and during the application of 100 μM DTT alone (1–5 min) and in combination with Ch-T (5–7 min) to a patch containing BK:LINGO2 channels. Panel *B* shows a summary of 5 to 7 experiments, in which DDT alone failed to alter the peak (*black circles*) or sustained (*purple diamonds*) currents. Furthermore, the effects of Ch-T were unaltered in the continuous presence of DTT (5–7 min). Panel *C* shows that DTT was unable to reverse the effects of Ch-T. BK:LINGO2 currents were evoked by a step to +160 mV and 200 μM Ch-T was applied from 1 to 4 min, before coapplication of Ch-T and DTT from 4 to 7 min. Panel *D* shows a summary of 4 to 7 experiments in which the inactivation was clearly abolished with Ch-T application (1–4 min), but it was not reversed by the coapplication of DTT and Ch-T from 4 to 7 min. BK, voltage-activated potassium; Ch-T, Chloramine T.

The effects of Ch-T were not prevented by pretreating patches with the reducing agent DTT (100 μM, Fig. 4, *A* and *B*), nor were they reversed by coapplication of DTT and Ch-T (Fig. 4, *C* and *D*) suggesting that the effects of Ch-T were not mediated *via* oxidation of cysteine residues. Consequently, we tested the possibility that other oxidizable residues, such as methionine, were involved.

**Figure 5:**
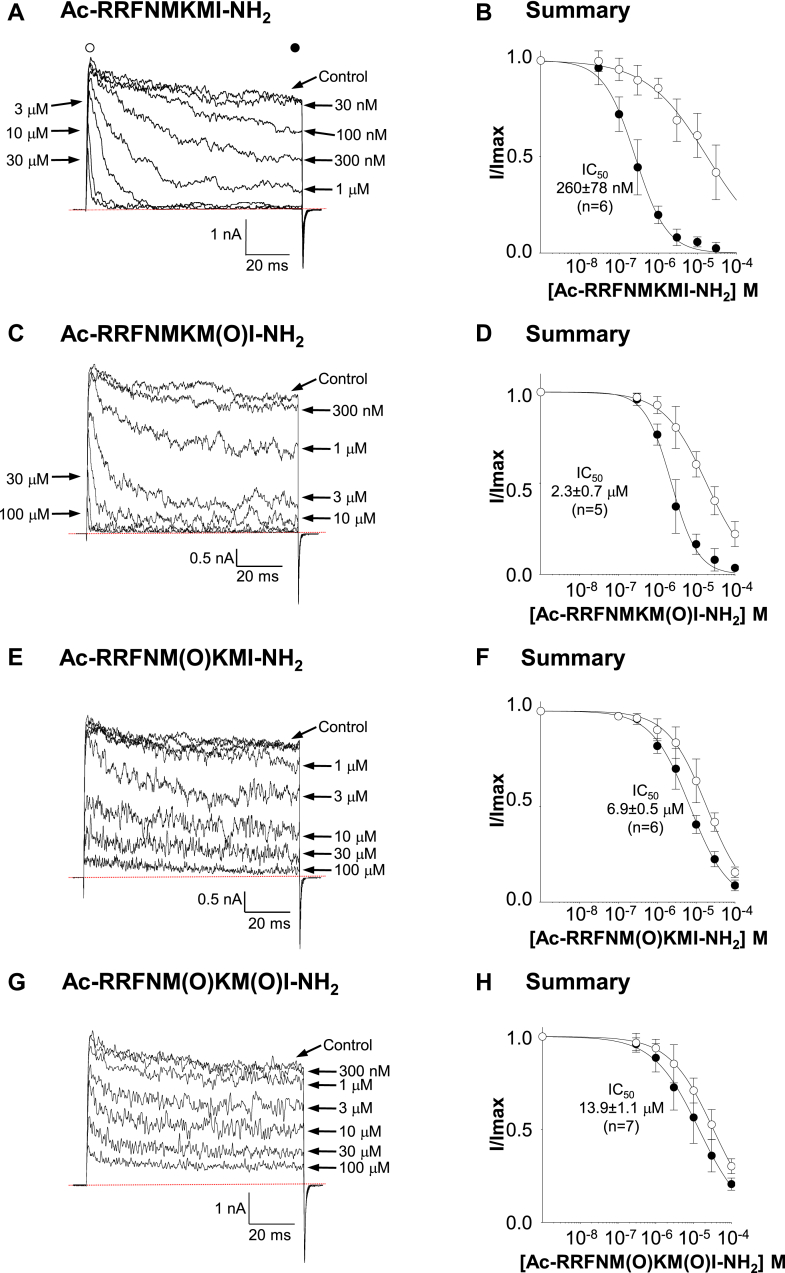
**Oxidation of methionine residues in LINGO2 tail peptides reduced their effects on BK channels.** HEK cell patches were bathed in 1 μM Ca^2+^ and sustained BKα currents were evoked by stepping to +160 mV for 100 ms from a holding potential of −60 mV, and the tails were evoked at −80 mV. The concentration-dependent effects of different peptides were examined, as shown in the Panels *A* and *B* for Ac-RRFNMKMI-NH_2_, Panels *C* and *D* for Ac-RRFNMKM(O)I-NH_2_, Panels *E* and *F* for Ac-RRFNM(O)KMI-NH_2_, and Panels *G* and *H* for the doubly oxidized peptide Ac-RRFNM(O)KM(O)I-NH_2_. The summary graphs in Panels *B*, *D*, *F*, and *H* were constructed for each peptide, and the half maximal inhibitory concentration (IC_50_) of each peptide was calculated from the current amplitudes measured in the first (*open symbols*) and the last (*black symbols*) 5 ms of each depolarizing pulse. The IC_50_ values on each graph were derived from the current amplitude recorded in the last 5 ms. Thus, the IC_50_ for Ac-RRFNMKM(O)I-NH_2_ (Panel *D*) was 2.3 ± 0.7 μM and was further increased in Panel F for the Ac-RRFNM(O)KMI-NH_2_ peptide to 6.9 ± 0.5 μM (*p* < 0.001, extra sum of squares F-test). These were ∼8 and ∼27 fold higher than the WT LINGO2 peptide shown in Panel B (260 ± 78 nM). As Panels *G* and *H* show, the ability of the double Met-O peptide Ac-RRFNM(O)KM(O)I-NH_2_ to cause inactivation was greatly reduced and its IC_50_ was ∼50 fold higher (13.9 ± 1.1 μM, *p* < 0.001, extra sum of squares F-test) than that observed with the WT LINGO2 peptide (Panel *B*). BK, voltage-activated potassium; HEK, human embryonic kidney.

**Figure 6:**
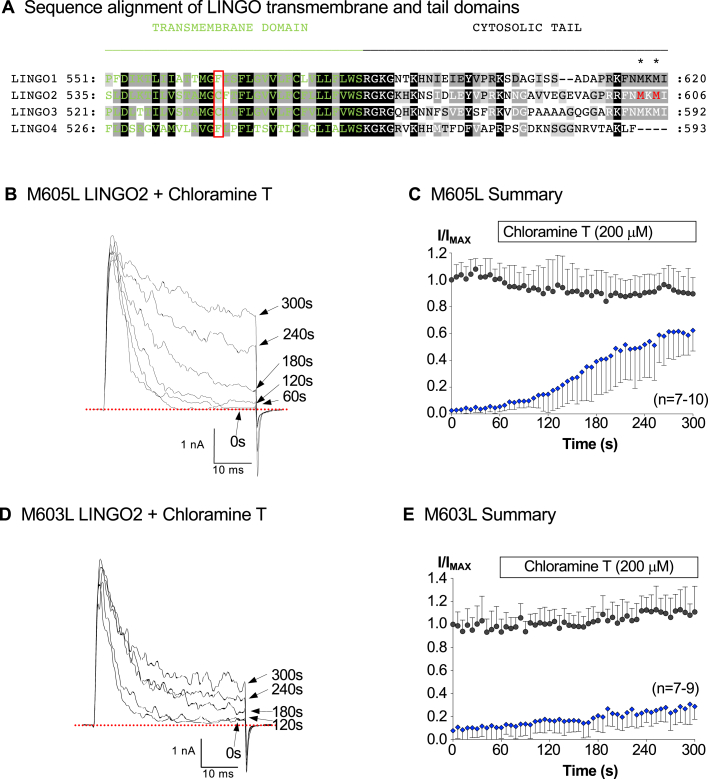
**LINGO2 M603 and M605 leucine mutants are partially resistant to chloramine-T oxidation**. Panel *A* shows a multiple sequence alignment for the LINGO1-4 subunits transmembrane and cytosolic tail domains, and the location of M603 and M605 are highlighted in *red text*. Panels *B* and *C* show a typical current record and summary data for the M605L mutant, respectively, before and during Ch-T treatment. Currents were evoked from M605L mutant by stepping to +160 mV in 100 nM Ca^2+^ and 200 μM Ch-T was applied after 60 s. As the summary data in *C* shows, it was able to increase the sustained current (*blue diamonds*) to ∼50% of the peak current amplitude at the end of 210 s application. This increase was significantly smaller compared to WT BK:LINGO2 (*p* < 0.001, Wilcoxin signed-rank test). Panel *D* shows currents elicited from the M603L mutant using the same protocol before and during application of Ch-T after 60 s, where it was clear that Ch-T was less effective at increasing the sustained current even after 4 min of continuous application. Panel *E* shows a summary of 7 to 9 similar patches in which the peak (*black circles*) and sustained current (*blue diamonds*) are recorded before and during Ch-T, which only increased the sustained current to ∼20%. BK, voltage-activated potassium; Ch-T, Chloramine T.

LINGO2 contains two methionine residues in its distal C-terminus (marked with asterisks in Fig. 6*A*), and we examined if their oxidation abolished inactivation. We have previously shown that the last eight residues of LINGO1 were sufficient to mimic the inactivation induced by the full-length LINGO1 protein (11). Therefore, we first synthesized a series of acylated amidated LINGO2 tail peptides comprised of the last eight residues of the LINGO2 protein. As Figure 5, *A* and *B* suggest, the Ac-RRFNMKMI-NH2 peptide caused inactivation, the rate of which was concentration-dependent, and the summary data in Figure 5*C* shows the mean reduction in current amplitude measured in the first (white circles) and last 5 ms (black circles) of the depolarizing pulse. The concentration of the peptide that produced half maximal block in current amplitude recorded in the last 5 ms of a pulse (IC_50Last5ms_) to +160 mV was 260 ± 78 nM (95% Confidence Intervals (CI) 223–303 nM, n = 6). Similar effects were observed with the RRFNMKM(O)I peptide, in which the methionine equivalent to M605 in the full-length peptide was oxidized. However, the rate of inactivation was slower compared to that observed with the WT peptide (compare currents in 1 μM of each peptide in Fig. 5, *A* and *C*), and the IC_50Last5ms_ was increased (2.3 ± 0.7 μM, 95% CI, 2.0–2.7 μM, *p* < 0.001, n = 5, extra sum of squares F-test), suggesting that its affinity for BK channels was reduced. Application of the RRFNM(O)KMI peptide, in which the methionine equivalent to M603 was oxidized, had much less effect, as illustrated in Figure 5, *E* and *F*. Thus, the inactivation in the presence of 1 μM of this peptide was slower than that of the WT peptide and the IC_50Last5ms_ was increased ∼27 fold (to 6.9 ± 0.5 μM, 95% CI 6.2 μM – 7.8 μM) compared to the WT peptide (n = 6, *p* < 0.001, extra sum of squares F-test). Finally, we applied a peptide in which both methionine residues were oxidized (RRFNM(O)KM(O)I) and found that inactivation was practically abolished at all concentrations tested (Fig. 5, *G* and *H*). The IC_50Last5ms_ was increased ∼50 fold to 13.9 ± 1.1 μM (95% CI 11 μM – 17 μM)) compared to the WT tail peptide (*p* < 0.001, n = 7, extra sum of squares F-test).

We next tested if substitution of each methionine in the C-terminus (see Fig. 6*A*) with leucine could protect the full-length LINGO2 from oxidation induced by Ch-T. We chose leucine because its side chain is similar in size and hydrophobicity but is more resistant to oxidation than the sulphur-containing side chain of methionine. Figure 6*B* shows a typical current from an experiment in which the M605L mutation was introduced into the full-length LINGO2 protein and cotransfected with BK cDNA. The currents evoked by a step to +160 mV retained the typical inactivation characteristics of WT LINGO2 (Fig. 2*A*), but the inactivation was affected by Ch-T. Thus, after 60 s application of Ch-T to the M605L mutant, the sustained current was increased from 6 ± 4% to 15 ± 14% of the peak current amplitude (Fig. 6*C*, n = 10, *p* < 0.01, Wilcoxin signed-rank test). After an additional 90 s exposure to the oxidizing agent, sustained current amplitude increased further to 47 ± 25% (*p* < 0.01, Wilcoxin signed-rank test), but this was ∼50% less effective than it was on WT BK:LINGO2 currents (*p* < 0.001, Mann–Whitney test). As Figure 6*D* suggests, the M603L mutant was even more resistant to the effects of Ch-T treatment, as evidenced by the relatively small increase in the amplitude of the sustained currents, following its application. Summary data in Figure 6*E* show that the mean sustained current under control conditions still significantly increased from 10 ± 9% to 17 ± 9% and 20 ± 12% in the presence of Ch-T for 60 s and 150 s, respectively (*p* < 0.05, n = 9, Friedman’s test), but this effect was significantly smaller than that observed in the LINGO2 M605L mutant (*p* < 0.05, Mann–Whitney test). As shown in Fig. S5, the inactivation of this mutant was also much more resistant to the effect of epifluorescent illumination (n = 6), compared to the WT BK:LINGO2 currents shown in Figure 2*A*.

**Figure 7:**
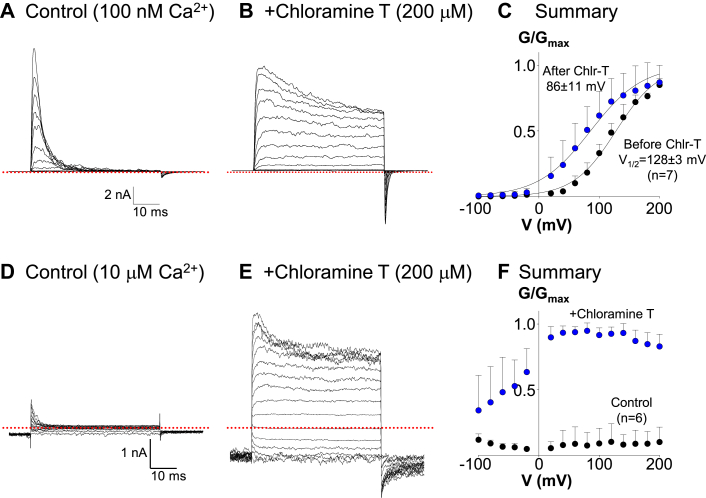
**Effects of Ch-T on BK:LINGO2 currents at different voltages.** Panels *A* and *B* show families of currents obtained from the same patches bathed in 100 nM Ca^2+^ before and after Ch-T treatment, respectively. Cells were stepped from −100 mV through to +200 mV for 50 ms in 20 mV increments. Ch-T (Panel *B*) increased current amplitude and practically abolished inactivation. Panel *C* shows summary GV curves before (*black circles*) and during (*blue circles*) Ch-T treatment, and data was normalized to currents obtained in 1 μM Ca^2+^ before Ch-T treatment. Data were fitted with a Boltzmann function (*solid lines*) to yield a V_1/2_ of 128 ± 3 mV before and 86 ± 11 mV after Ch-T treatment in seven patches. Panels *D* and *E* used the same voltage protocol as Panels *A* and *B*, but the [Ca^2+^] was 10 μM as evidenced by the almost complete absence of currents at any potential (Panel *D*). However, after Ch-T treatment (Panel *E*), the current amplitudes at all voltages were clearly increased, such that large inward currents were recorded at potentials negative to zero. A similar pattern was seen in six similar experiments summarized in Panel *F*, where the G/G_max_ was plotted after normalization of the currents to those obtained in Ch-T. It was clear that Ch-T treatment increased the G/G_max_ at all voltages in 10 μM Ca^2+^. For example, at −60 mV, application of Ch-T significantly increased the G/G_MAX_ from 0.06 ± 0.02 to 0.48 ± 0.27 (n = 6, *p* < 0.05, Wilcoxin signed-rank test). BK, voltage-activated potassium; Ch-T, Chloramine T; GV, conductance versus voltage.

We next examined the effects of Ch-T on BK:LINGO2 currents recorded at different voltages and in varying [Ca^2+^]_i_. Figure 7*A* shows a family of currents evoked by stepping from −100 mV to +200 mV in 20 mV increments for 50 ms from a holding potential of −100 mV and recorded with 100 nM Ca^2+^ bathing the cytosolic surface of the patch. Figure 7*B* shows the currents recorded from the same patch after Ch-T had its maximal effects and demonstrates that inactivation was practically abolished, as evidenced by the amplitude of the currents at the end of the depolarizing pulses. The summary conductance versus voltage (GV) curves in Figure 7*C* were constructed from seven similar experiments conducted before (black symbols) and after applying Ch-T (blue symbols) for 2 min, which shifted the half maximal activation voltage from 128 ± 3 mV to 86 ± 11 mV. Figure 7*D* shows currents recorded from a different patch, which was bathed in 10 μM Ca^2+^ at its cytosolic surface and stepped from −100 mV to +200 mV in 20 mV steps. Under these conditions, inward currents were recorded at negative potentials and small, inactivating, outward currents were recorded at more positive potentials. However, after treatment with 200 μM Ch-T (Fig. 7*E*), the current amplitude was increased at all voltages recorded, and inactivation was practically abolished. Figure 7*F* shows summary GV data from six similar experiments recorded in 10 μM Ca^2+^ in the absence (black symbols) and presence (blue symbols) of Ch-T and illustrated that oxidation led to large increases in G across the voltage range.

To examine if the steady-state voltage-dependent inactivation was altered, we used a double pulse protocol (inset Figure S-4) before and after removal of inactivation with Ch-T. As Fig. S4, *B* and *C* demonstrate, no time- or voltage-dependent inactivation was observed after Ch-T treatment.

**Figure 8:**
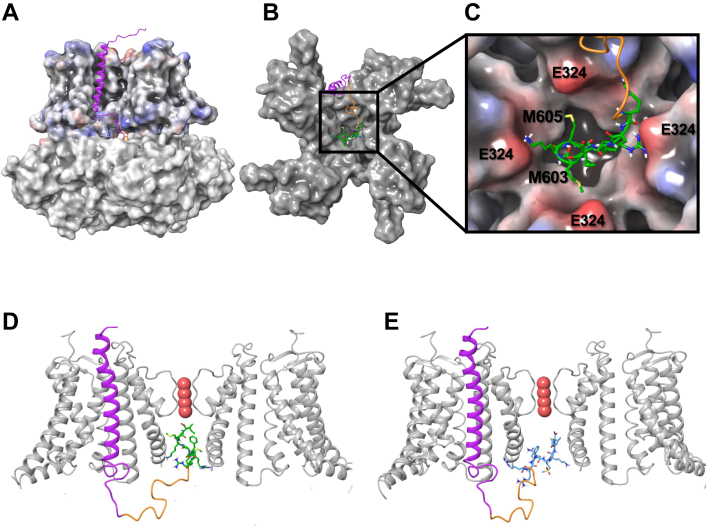
**Representation of the BK:LINGO2 channel complex from molecular docking.** Panel *A* shows a side view of the overall complex in which the BK channel is shown in the surface representation with the transmembrane part of the channel, colored based on electrostatics and the intracellular side on the *white surface*. LINGO2 is shown in the *cartoon* representation. Panels *B* and *C* show the overall and close-up views, respectively, of the complex from the intracellular side. The transmembrane helix, the peptide linker, and the 8-residue C-terminus of LINGO2 are in *purple*, *orange*, and *green*, respectively. The terminal 8-residue peptide is in *green sticks*. Panel *D* shows the side view of the complex, but two subunits of the channel were removed for clarity. The BK channel and LINGO2 are shown in the cartoon representation, and only the side chain and backbone of the terminal eight LINGO2 residues are shown. The K^+^ ions are in the space-filling representation. Panel *E* shows the side view of the docking complex when residue M603 was oxidized and is shown as *blue sticks*. BK, voltage-activated potassium.

In an attempt to explain how oxidation might lead to the removal of inactivation and in the absence of published BK:LINGO2 structures, we performed docking studies of LINGO2 to the BK channel, as shown in Figure 8. Initially, the 8-residue peptide and the transmembrane helix of LINGO2 were docked to the BK channel separately. From the flexible docking of the peptide, the final peptide docking pose in the BK channel was chosen to be able to link to the LINGO2 helix. The LINGO2 helix model was taken from the AlphaFold database (https://alphafold.ebi.ac.uk/), and its position in the channel was identified by rigid-body docking. LINGO2 was docked similarly to the auxiliary β4 protein in the crystal complex with the BK channel (21). Next, both parts of LINGO2 were linked by a flexible loop, which was predicted by a loop prediction approach with consideration of its interactions with the BK channel and is shown in Figure 8*A*. As it suggests, the tail of LINGO2 gained access to the BK channel pore through a side portal between the transmembrane domain and the intracellular RCK domains of the BK channel. The LINGO2 tail docked centrally in the BK channel pore as shown in Figure 8*B*. The optimized BK:LINGO2 channel complex showed that the positively charged residues of the LINGO2 C-terminal peptide formed ionic interactions with the E324 residues of the BK subunits, whereas hydrophobic residues of the peptide sat in the hydrophobic pockets of the pore. Interestingly, the docking suggested that while M605 was buried deep in a hydrophobic pocket of the pore, M603 had a more solvent-exposed position (Fig. 8*C*). These configurations were also preserved when the M603L and M605L mutants were modeled (Fig. S6). A side-on view of the optimized BK:LINGO2 complex is presented in Figure 8*D* and illustrates that the eight terminal residues of the LINGO2 tail, shown in green sticks, penetrated deep into the pore and the I606 residue sat just below the selectivity filter. This could help provide a structural explanation of how the C-terminus of LINGO2 occluded the BK channel pore, but structural studies will be required to confirm this. Interestingly, as the blue sticks shown in Figure 8*E* suggest, when the M603 oxidized form of the peptide was docked, it failed to penetrate as deeply into the channel pore.

## Discussion

The results of the current study demonstrate that when BK channels were coexpressed with LINGO2, the resultant currents inactivated rapidly and activated at more negative potentials than BKα subunits, similar to the effects of LINGO1 on BK channels (11). This is not surprising as LINGO1 and LINGO2 look qualitatively similar, sharing 61% identity and 77% homology (22). However, there are a number of distinguishing features between BK:LINGO1 and BK:LINGO2 currents. Firstly, coexpression of LINGO2 with BK channels did not significantly reduce the amplitude of the inactivating currents (Fig. S2), suggesting that this subunit does not alter plasmalemmal expression of BK channels, in contrast to that observed previously with LINGO1 (11). Secondly, the activation V_1/2_ for BK:LINGO2 currents was ∼130 mV in 100 nM Ca^2+^, compared to 100 mV for BK:LINGO1 under identical recording conditions (11). Thirdly, BK:LINGO2 currents inactivated more slowly than BK:LINGO1 currents (inactivation time constant at +100 mV of ∼10 ms for BK:LINGO2 and ∼5 ms for BK:LINGO1). Similarly at +200 mV, LINGO2 inactivated with a τ of 4 ms compared to ∼2 ms observed in BK:LINGO1 channels (11). Fourthly, when steady-state inactivation was investigated with a double pulse protocol, we found that BK:LINGO2 channels half maximally inactivated at voltages ∼30 mV more positive than those previously recorded in BK:LINGO1 channels. Thus, in the present study we found that the inactivation V_1/2_ for BK:LINGO2 was +35 mV in 100 nM Ca^2+^ and this shifted to ∼-40 mV when the cytosolic [Ca^2+^] was increased to 1 μM. Given that the inactivation observed with LINGO1 and LINGO2 appears to be due to open channel block, we speculate that the differences in the above biophysical properties are due to the differences in activation V_1/2_ observed between BK:LINGO1 and BK:LINGO2.

Li *et al.* (23) demonstrated that a phenylalanine residue (F273) in the transmembrane domain of the leucine-rich repeat-containing protein, γ3, was responsible for the shift in BK activation V_1/2_. Interestingly, LINGO1 has a phenylalanine (the red box in Fig. 6*A*) at the equivalent position to F273 of the γ3 subunit, whereas LINGO2 does not. This may at least partly explain why BK:LINGO2 channels do not activate as negatively as BK:LINGO1 channels. Another interesting observation is that LINGO2 has three positively charged residues in the juxta-membrane region, whereas LINGO1 only has two. Li *et al.* (23), demonstrated that positively charged juxta-membrane residues contributed to the shift in activation V_1/2_ observed with γ3 subunits; therefore, it will be interesting to examine if this accounts for the differences in V_1/2_ between BK:LINGO1 and BK:LINGO2 currents.

Another important observation on BK:LINGO2 currents was that the inactivation waned rapidly in patches exposed to epifluorescent illumination. The ability of illumination to remove inactivation was practically abolished when GFP was omitted from the transfection. A number of studies have demonstrated that GFP can alter the redox state of cells (24, 25), and its illumination has been shown to generate reactive oxygen species (26, 27), which can oxidize proteins, including ion channels, and modulate their function (28, 29, 30, 31, 32, 33, 34, 35, 36). For example, Pooler (28) demonstrated that photo-oxidant stress slowed inactivation of Na^+^ currents, and Ciorba *et al.* (31) showed that Ch-T reduced the inactivation of *Shaker* C/B K^+^ channels but failed to alter the inactivation of the *Shaker* B splice variant. Ciorba *et al.* (31) also demonstrated that oxidation mediated its effects *via* methionine rather than cysteine residues in the N-terminus of the *Shaker* C/B channel tail. They also showed that the *Shaker* B splice variant contained only one methionine residue (M1) in its amino terminus ball domain (M1), compared to the *Shaker* C/B splice variant, which, like LINGO2, contained two methionines (M1 & M3) in its tail. Our results suggest that the removal of inactivation by Ch-T was not due to an effect on cysteine residues, given the insensitivity of the responses to DTT treatment. Instead, a number of lines of evidence support the idea that Ch-T mediated its effect *via* oxidation of M605 and M603 in the C-terminus of LINGO2. Firstly, synthetic peptides containing Met(O) at either positions, showed a decreased rate of inactivation and a reduced affinity for the BK channels. Furthermore a peptide containing Met(O) at both positions showed an ∼50-fold reduction in IC_50_ and time-dependent inactivation was practically abolished. Secondly, replacement of either methionine with leucine in the full-length LINGO2 protein markedly reduced the effects of oxidation. The M603L mutant was particularly resistant to CT-T treatment and the effects of illumination (Fig. S5), suggesting that this residue may play a more prominent role in this response. The modeling data shown in Figures 8 and S6 are consistent with this idea and potentially provide a structural basis for these observations. Thus, in the absence of oxidation, the C-terminus of the LINGO2 protein docks deep in the BK channel pore and M605 is buried in a hydrophobic region, which would reduce its ability to be modified by oxidizing stimuli. In contrast, the M603 residue is exposed to the aqueous environment of the pore and thus could be more easily oxidized. Such an arrangement is consistent with the different rates of removal of inactivation by Ch-T shown in Figure 6, *C* and *E*. The resultant oxidation of either of these methionine residues would reduce their lipophilicity and disrupt any lipophilic interactions in the pore, which presumably reduces the affinity of the inactivating particle and consequently decreases inactivation. Figure 8*E* shows an example of the consequences of having M603 oxidized, where it is clear that the last eight residues (blue sticks) of the tail peptide fail to dock as deeply in the pore, compared to the WT LINGO2 tail shown in Figure 8*D*. This more surfaced pose of the oxidized LINGO2 tail may provide a structural hypothesis for the lack of channel inactivation observed during oxidation, although this will require experimental confirmation in future structural studies.

The sensitivity of BK:LINGO2 channels to oxidation may provide an additional mechanism for dynamically modulating BK channel activity. The functional consequences of such modulation are illustrated in Figure 7, *D*–*F*, where Ch-T in 10 μM Ca^2+^ significantly increased the open probability of BK:LINGO2 channels, even at physiological potentials. Interestingly, in contrast to early reports that LINGO proteins were exclusively expressed in the CNS, recent immunohistochemical studies have demonstrated that LINGO proteins are also expressed on human intestinal epithelial cells (LINGO2, (37); LINGO3, (38)), endometrial smooth muscle (LINGO2, (39)), and in the human respiratory system (LINGO1, (39); LINGO3, (38)). Given that LINGO1-3 proteins possess the same pattern of two methionines in the last four residues of their C-termini (Fig. 6*A*), it is possible that they all can be modulated to various extents by oxidation. We speculate that this may be particularly important in the tissues exposed to a highly oxidizing environment such as the epithelial cells of the respiratory system. We surmise that the oxidation of BK:LINGO1-3 channels could dynamically regulate BK channel function to increase their open probability, but this will require experimental confirmation.

In conclusion, we present evidence that LINGO2 interacts with BK channels to shift their voltage of activation negatively and induce inactivation, which is subject to regulation by oxidation of two methionine residues in the C-terminus tail of LINGO2.

## Experimental procedures

### Solutions

All excised patch experiments were performed at 37 °C in 140 mM symmetrical K^+^ solutions which contained 140 mM KCl, 10 mM glucose, 10 mM Hepes, and either 1 mM ethylene glycol-bis(β-amino ether)-N,N,N**′,N′-tetraacetic acid** (EGTA (for free [Ca^2+^] 100 nM to 300 nM) or 1 mM hydroxyethylethylenediaminetriacetic acid (HEDTA for free [Ca^2+^] 1 μM to 10 μM) and the pH of all solutions was adjusted to 7.2 with KOH. These solutions were made with double-distilled, deionized, filtered water from a MilliQ water purification system. The pipette solution contained 100 nM free Ca^2+^. We used “Chelator” (https://www.ru.nl/animal/research/chelator/) to calculate the total amount of Ca^2+^ required, as per Schoenmakers *et al.*, (40) and free Ca^2+^ concentrations were checked with a Ca^2+^ sensitive electrode.

During experiments, the dish containing HEK cells was superfused with Hanks solution, which contained (in mM) 129.8 Na^+^, 5.8 K^+^, 135 Cl^-^, 4.17 HCO_3_^-^, 0.34 HPO_4_^2-^, 0.44 H_2_PO_4_^-^, 1.8 Ca^2+^, 0.9 Mg^2+^, 0.4 SO_4_^2-^, 10 glucose, 2.9 sucrose, and 10 Hepes, and its pH was adjusted to 7.4 with NaOH. In addition, the patch under study was continuously superfused by means of a close delivery system consisting of a pipette (tip diameter 200 μm) placed approximately 300 μm away from the cell. This could be switched, with a dead-space time of around 5 s, to a solution containing a drug.

### Electrophysiology

Electrodes were pulled from Corning borosilicate glass (1.5 mm O.D. × 0.86 mm I.D.) using a Sutter P-97 pipette puller and fire polished using a Narashige MF 83 microforge. Pipettes had resistances of 2 to 5 MΩ when filled with recording solutions and series resistance was compensated by up to 80%. Standard single-channel patch clamp recording methods were used. Voltage clamp commands were delivered *via* an Axopatch 200B patch clamp amplifier (Axon Instruments) connected to a Digidata 1322A AD/DA converter (Axon Instruments) interfaced to computers running pClamp software (Axon Instruments). Data was acquired at 100 KHz and filtered at 2 KHz or 5 KHz. Patches were held at either −60 mV or −100 mV and depolarized in 20 mV increments to 200 mV. Residual capacitance and leakage currents were subtracted using either a P/4 protocol or offline by manual leak subtraction.

### Illumination

We used either a cool LED PE100 470 nM LED (CoolLED) light source (at 75% intensity) attached *via* a fibre optic guide to the rear port of a Nikon Diaphot microscope, through a standard FITC filter set, to a Nikon Plan 40/0.55 objective, or *via* a Nikon Mercury Lamp (C-SHG1) through the rear port of a Nikon Eclipse TE2000S microscope and passed through a standard FITC filter set to a Nikon Pan Fluo 40×/0.6 objective. Inactivation of BK:LINGO2 currents in excised patches was abolished within 4 min of illumination.

### Ion channel cloning and mutagenesis

The human LINGO2 transcript used [NM_001354575.2] was untagged in a pcDNA 3.1 vector (VectorBuilder). The α subunit of the rabbit BK channel was isolated from rabbit urethral smooth muscle and cloned using the pcDNA TOPO DNA cloning kit (Life Technologies). The identifiesd transcript corresponded to the ZERO variant of mouse BKα and to variant 2 (NM_002247.3) of human BKα. We used 100 ng ml^−1^ cDNA for BKα only transfections, whereas a 100:500 ng ml^−1^ cDNA ratio was used for BKα:LINGO2 and BKα:LINGO1 cotransfections for inside-out patch recordings. GFP (150 ng ml^−1^) was included in all transfections except those used for Figure 2, *C* and *D*. cDNA was transiently transfected into HEK cells using lipofectamine. Site-directed mutagenesis used the Phusion kit (Thermo Fisher Scientific) method (41) and all mutations were confirmed by sequencing.

HEK293 cells were cultured in Dulbecco's modified Eagle's medium + MEM media containing 10% fetal bovine serum and 1% Penicillin-Streptomycin antibiotics at 37 °C, and 95% humidifying incubator containing 5% CO_2_. Subculturing was done by 0.05% Trypsin-EDTA solution.

### Insertion of HA sequence into LINGO2

A PCR strategy was used to incorporate the coding sequence of the HA epitope YPYDVPDYA into full-length LINGO2 between residues 27 and 28 with the HA sequence shown in bold - STIG***YPYDVPDYA***CPAR. Oligonucleotides were designed coding for the HA sequence and a small complementary section of LINGO2 sequence to insert the HA tag downstream of the signaling sequence. Reaction mixes containing template, Phusion Pfu (Thermo Fisher Scientific), GC buffer, dNTPs, DMSO, and either forward or reverse primers in separate tubes were cycled in a PCR machine for 10 cycles of 98 °C − 10 s, 65 °C − 30 s, and 72 °C − 3 min 30 s. Forward and reverse reaction mixes were then combined for 20 cycles of PCR using the same reaction conditions. PCR products were treated with DpnI (New England Biolabs) and transformed into XL10 competent cells. Resultant colonies were then DNA extracted and the plasmids sequenced in both directions to check for HA insertion and sequence integrity.

LINGO2-HA-fwd: TAC CCA TAC GAT GTT CCA GAT TAC GCT tgc ccc gct cgc t.

LINGO2-HA-rev: AGC GTA ATC TGG AAC ATC GTA TGG GTA gcc aat ggt gga

### Generation of the tagged BK channel

The ZERO variant of the BK channel with an N-terminal Flag-epitope and C-terminal–HA epitope (Flag-ZERO–HA) was constructed in pcDNA3 as previously described by Chen *et al* 2005 (42). The N-terminal sequence, thus, begins immediately upstream of MDALI start site with the sequence M***DYKDDDDK***MDALI.. (Flag-sequence in bold italic). The HA tag at the C-terminus replaced the normal stop codon of the ..REVEDEC tail thus the C-terminus sequence is .REVEDEC***YPYDVPDYA∗ (-HA sequence*** in bold italic).

To engineer the Flag–ZERO-myc construct, a short sequence of the C-terminal tail spanning the internal SfiI site and a NotI site in the vector backbone was synthesized de novo (Twist Bioscience) and ligated into the Flag-ZERO–HA construct to replace the HA tag with a C-terminal–myc tag. The C-terminal amino acids of Flag-ZERO–myc was, thus, …REVEDEC***EQKLISEEDL∗.***

### Electrophysiology curve fitting and statistics

*G* was derived from peak currents according to Ohm’s law

[1] $$G=\frac{I}{\left(V-EK\right)}$$

where, *E*_K_ = 0 mV in symmetrical [K^+^]. Summary data was expressed as the mean ± SEM. *G–V* relationships were fitted with the Boltzmann equation

[2] $$\frac{G}{G_{MAX}}=\frac{1}{1+e\left(\left(Vm-V1/2\right)/S\right)}$$

where, *V*_1/2_ is the voltage of half-maximum activation, *S* is the slope of the curve, V_*m*_ is the test potential, G is the conductance, and G_*max*_ is the maximal conductance. For BK constructs, data from each patch was normalized to the peak conductance measured in either 1 μM or 10 μM Ca^2+^ to obtain G_max_, and curves were constrained to the G_*max*_ value obtained in this. In full-length BKα:LINGO2 channels, currents were normalized to the peak G_*max*_ recorded in 1 μM Ca^2+^ or 100 nM Ca^2+^.

Inactivation curves were fitted with a similar Boltzmann function:

[3] $$I/I_{\max}=1/\left\{1+\exp\left[\pm \left(V_{1/2}-V_{c}\right)/K\right]\right\}$$

where, *I* was the current recorded at the test step, *I*_max_ was the maximal current recorded, *V*_c_ was the conditioning potential (see RESULTS), and *K* was the slope factor.

Concentration–effect data were fitted with a Hill–Langmuir equation:

[4] $$I/I_{control}=1/\left\{1+10^{ˆ}\left(\log\left[Drug\right]-\log\;IC_{50}\right)\right\}$$

where, *I* was the current recorded in the drug, *I*_control_ was the current in the absence of drug, IC_50_ was the half-maximal effective concentration, and [Drug] was the concentration variable.

### Statistical tests

All data is presented as mean ± SD and was analyzed with Prism software (GraphPad). Individual data points are also shown in bar charts. Statistical differences were assessed using either paired or unpaired nonparametric tests (Wilcoxin signed-rank test, Mann–Whitney test), or the extra sum of squares F-test, as appropriate. Comparisons of three or more nonparametric data sets used Friedman’s test followed by Dunn’s test for multiple comparisons, whereas ANOVA was used for three or more parametric data sets. *p* < 0.05 was taken as significant.

### Molecular modeling

The structure of the Ca^2+^ bound human BK channel in complex with the β4 subunit with PDB code: 6V22 (21) was used for protein–peptide docking. The eight C-terminus residues of LINGO2 in the WT, M603 oxidized form, M603L, and M605L were first docked to the BK channel opening using the Glide module of Schrodinger software (Release 2020–1; (43)). The protein and peptide structures were prepared with the protein preparation module of Schrodinger software using a standard protocol. Residues involving L312, F315, S317, V319, P320, E321, I323, and E324 were selected as the center of the docking box. Docking poses were evaluated with the Glide docking score. The frequency of docking pose and the possibility of linking the peptide with the rest of LINGO2 were considered in the final docking pose selection. The N-terminal transmembrane helical part of LINGO2 was taken from the AlphaFold database (44) and docked to the BK channel, using the HEX protein–protein docking program (45) with a default protocol. The peptide linker between the transmembrane helix and the C-terminal 8-residue peptide was built taking into account the architecture of the BK channel using the Prime module of Schrodinger (46). Next, the transmembrane part of LINGO2 was manually connected to the 8-residue peptide, and the entire structure was optimized. Minimization of docking complexes was carried out with the MacroModel module of Schrodinger software. A default protocol of minimization in the implicit solvent with a distance-dependent dielectric constant was used to obtain the final complexes. The hydrogen bonds of the helices were constrained to avoid the structure deformation. The OPLS2005 force field was used in all calculations. The 3D images were created in Maestro 2020 to 1.

### Coimmunoprecipitation assay

HEK293T cells were seeded out at 4 × 10^6^ in a 10 cm dish in complete media (Dulbecco's modified Eagle's medium; 10% fetal calf serum and 1% PenStrep) and incubated overnight at 37 °C in 5% CO_2_. The next day, Flag-Zero–myc and HA-LINGO2 cDNA (each at 441 ng ml^−1^ to maximize protein expression) were cotransfected into the cells using the TransIT-293 transfection reagent (3:1; carrier: DNA) (Mirus Bio). The transfected cells were incubated for 72 h at 37 °C in 5% CO_2_.

For harvesting lysates, cell-conditioned media were aspirated, and cells were scraped into 500 μl ice-cold immuno-precipitation (IP) lysis buffer (20 mM Tris, 150 mM NaCl, 0.2% NP-40, 10% glycerol, pH 7.0, plus protease inhibitor cocktail and phosphostop tablets). The cell suspension was incubated on ice for 15 min with intermittent vortexing, before centrifugation (4 °C, max speed for 10 min) and collection of the supernatant. The lysates were quantified by bicinchoninic assay (BCA) and kept on ice until IP.

For the immunoprecipitation, the Dynabeads Protein G immunoprecipitation kit (Invitrogen, 10007D) was used, following manufacturer’s instructions. Briefly, 1 mg beads were transferred to fresh Eppendorf’s, and storage buffer was aspirated using magnet. Antibody binding buffer was added (200 μl) to each tube, followed by 8 μg antibodies (anti–Flag-M2; Sigma F3165 *or* anti-mouse IgG1; BioLegend 401,402) and incubated on a rotator for 1 h at room temperature (RT). Conjugated Ab-Bead complexes were then washed 3× in wash buffer before storing in 50 μl binding buffer. Lysates were added to the bead mixtures at 1 mg per tube. Inputs were retained in Eppendorf’s at 20 μg. The IP tubes were filled up to 750 μl final volume using binding/wash buffer and rotated for 1 h at RT. Post incubation, the beads were washed 3× using the wash buffer and resuspended in elution buffer with lithium dodecyl sulfate (4×) (final volume 25 μl). Input and IP samples were then denatured at 94 °C for 10 min before separation by SDS-PAGE.

For immunoblotting, lysates were separated using 4 to 12% NuPage BT gel for 1 h at 200 V, transferred onto nitrocellulose using iBlot2 (25V for 8 min), transfer confirmed by Ponceau S staining, membrane was blocked in 5% milk for 1 h at RT and the membrane incubated in anti-HA antibody (CST; 3724) overnight on a roller at 4 °C. For imaging, the membranes were washed 3× in TBST before incubating in anti–rabbit-800 secondary Ab (LiCor; 926–32211) for 1 h at RT in a dark box. The membrane was washed 3× in TBST before imaging using LiCor Odyssey imager.

### Reagents

Fluorenylmethyloxycarbonyl (Fmoc) amino acid derivatives, di-isopropylethylamine, trifluoracetic acid, pyridine, phenol, ethanedithiol, thioanisole, (1-cyano-2-ethoxy-2-oxoethylidenaminooxy)dimethylamino-morpholino-carbenium hexafluorophosphate (COMU), and rink amide 9H-fluoren-9-ylmethyl N-[[4-[2-[bis(4-methylphenyl)methylamino]-2-oxoethoxy]phenyl]-(2,4-dimethoxyphenyl)methyl]carbamate (MBHA) resin (0.5 mmol/g) were purchased from Sigma-Aldrich. Caplugs Evergreen 5-inch chromatography columns, piperidine, DMF, and all other solvents (HPLC grade) were purchased from Thermo Fisher Scientific.

#### LINGO2 tail peptide synthesis

All peptides were prepared manually using standard Fmoc strategy on a rink amide MBHA resin (47). The resin (100 mg) was swollen in dimethylformamide (DMF) (2 ml) before use, in a polypropylene-fritted chromatography column. Deprotection of the resin was effected by shaking in a solution of piperidine in DMF (1:4 v/v) for two periods of 25 min. The resin was then washed with DMF (x4), DCM (x4), and DMF (x4). For amino acid coupling, Fmoc-protected amino acid derivatives (3 equiv.) were activated with di-isopropylethylamine (3 equiv.) and COMU (3 equiv.) (48) in DMF, loaded onto the resin, and shaken for 45 min. The resin was then washed with DMF (x4), DCM (x4), and DMF (x4). Deprotection of the N-terminus of the growing peptide chain was accomplished by two 25-min periods of shaking in a solution of piperidine in DMF (1:4 v/v). Successful coupling and deprotection steps were confirmed using the ninhydrin test (49, 50). N-terminus capping was carried out using a solution of acetic anhydride:pyridine:DMF (2:2:6 v/v) for 2 h. Global deprotection and cleavage of the peptide from the resin was performed using a cocktail containing trifluoracetic acid/water/phenol/ethanedithiol/thioanisole (82.5/5/5/2.5/5 v/v) for 2 h under a blanket of nitrogen. Peptides were precipitated in ice-cold ether and centrifuged at 3000 rpm for 10 min. The resulting solid was isolated by filtration, washed several times with ether, dried under vacuum, and stored at −20 °C. Peptides were analyzed by reverse-phase–HPLC and high-resolution mass spectrometry (HRMS). **Ac-RRFNMKMI-NH**_**2**_. White Solid. (25 mg). HRMS (ESI-TOF): m/z [M + H]^+^ Calcd for C_49_H_86_N_17_O_10_S_2_ 1136.6185. Found 1136.6165. **Ac-RRFNM(O)KM(O)I -NH**_**2**_. White Solid. (45 mg). HRMS (ESI-TOF): m/z [M + H]^+^ Calcd for C_49_H_86_N_17_O_12_S_2_ 1168.6078. Found 1168.6083. **Ac-RRFNM(O)KMI-NH**_**2**_**.** White Solid. (42 mg). HRMS (ESI-TOF): m/z [M + H]^+^ Calcd for C_49_H_86_N_17_O_11_S_2_ 1152.6134. Found 1152.6124. **Ac-RRFNMKM(O)I-NH**_**2**_. White Solid (32 mg). HRMS (ESI-TOF): m/z [M + H]^+^ Calcd for C_49_H_86_N_17_O_11_S_2_ 1152.6134. Found 1152.6115.

## Data availability

All data are available upon request to MA Hollywood at mark.hollywood@dkit.ie.

## Supporting information

This article contains supporting information (Figs. S1 to S6).

## Conflict of interest

The authors declare that they have no conflicts of interest with the contents of this article.
