# Supplementary material for: Oxidation modulates LINGO2-induced inactivation of large conductance, Ca2+-activated potassium channels
Source: J Biol Chem. 2023 Feb 2;299(3):102975. doi: 10.1016/j.jbc.2023.102975 (PMC10020666; doi:10.1016/j.jbc.2023.102975)
Supplement: Supplemental figures [file mmc1.pdf]

A. BKα:LINGO2 recovery from inactivation

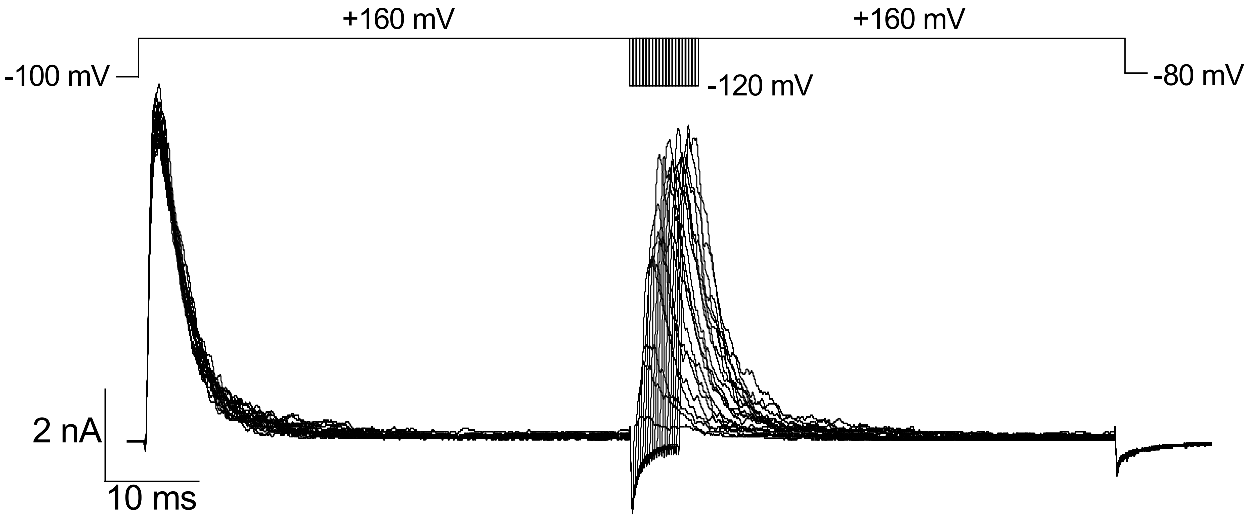

B. Summary data

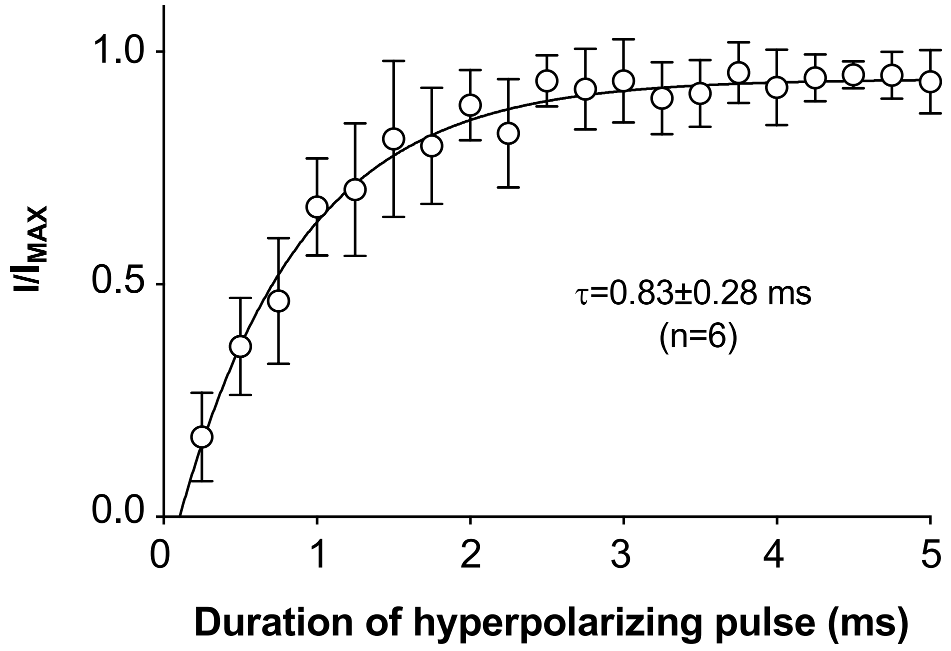

Figure S-1. BKα:LINGO2 currents recover rapidly from inactivation. Patches were bathed in 100 nM Ca^{2+} and stepped to +160 mV for 50 ms to induce complete inactivation. They were then hyperpolarized to -120 mV for 0-5ms in 0.25ms increments before stepping to +160 mV for 50 ms. Panel A shows a typical experiment and panel B shows a summary from 6 similar experiments in which the recovery of inactivation was plotted against time and fitted with an exponential (solid line).

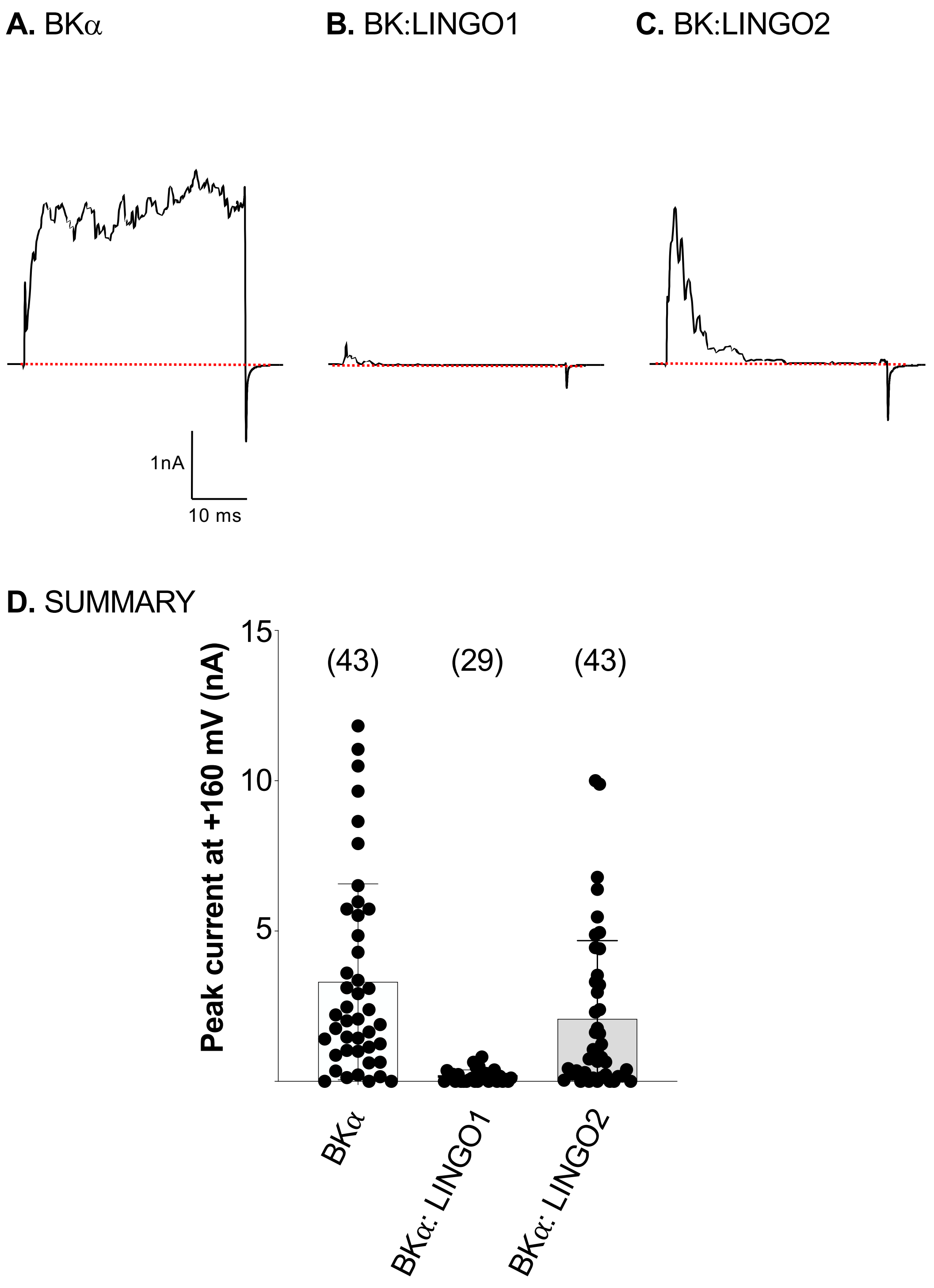

Figure S-2: *Plasmalemmal expression of A. BKα, B. BKα:LINGO1 and C. BKα:LINGO2 channels.* All patches were obtained with 5 MΩ pipettes and currents were evoked by a step to +160 mV for 40 ms, with 100 nM Ca^{2+} bathing the cytosolic surface of the patches. Tail currents were evoked at -80 mV. Panel D shows that the mean current of BKα patches was significantly greater than BKα:LINGO1 but not significantly different to BKα:LINGO2 was (ANOVA).

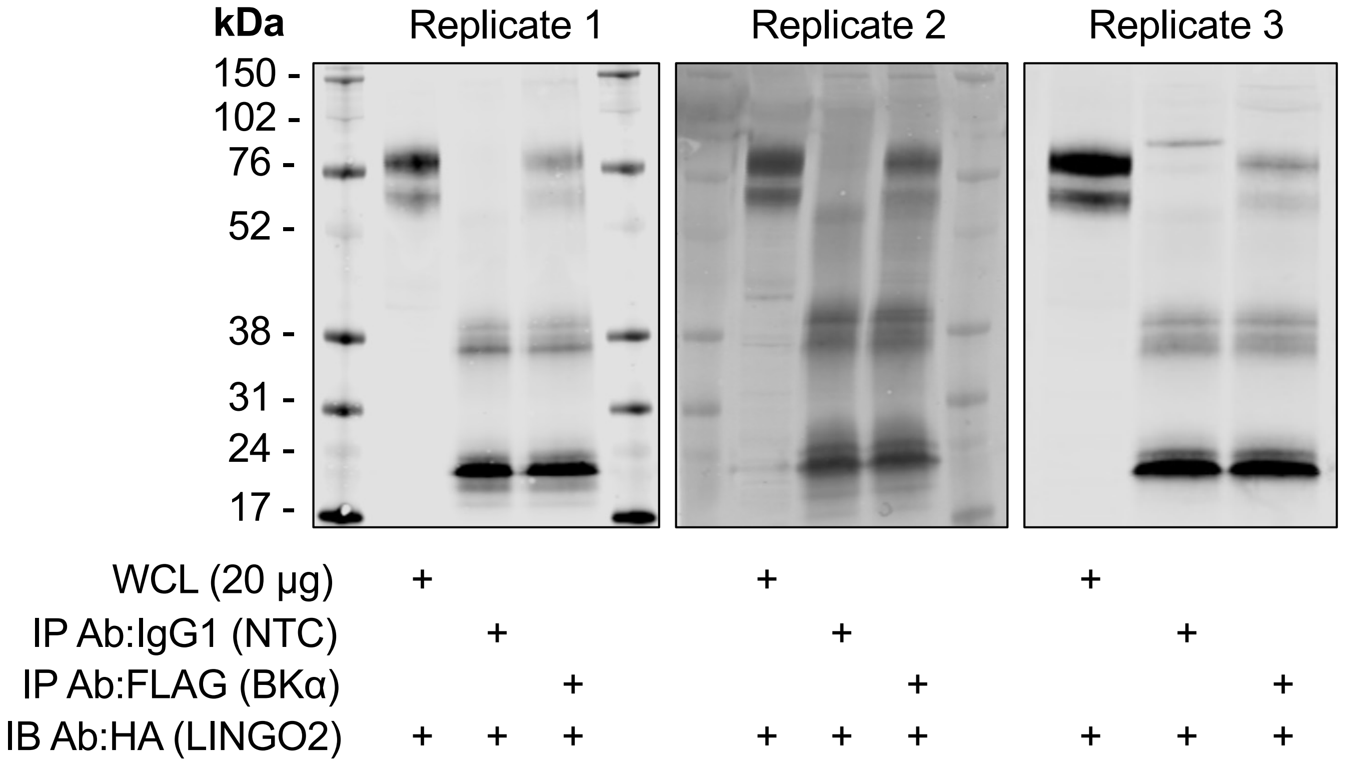

Figure S-3. Co-immunoprecipitation of whole cell lysates (WCL) from HEK293T cells demonstrates an interaction between FLAG-tagged BKα and HA-tagged LINGO2 proteins. Lysates were immunoprecipated with either anti-IgG1 or anti-FLAG antibodies and immunoblotted with an anti-HA antibody to detect LINGO2. This experiment was repeated in a total of 3 samples.

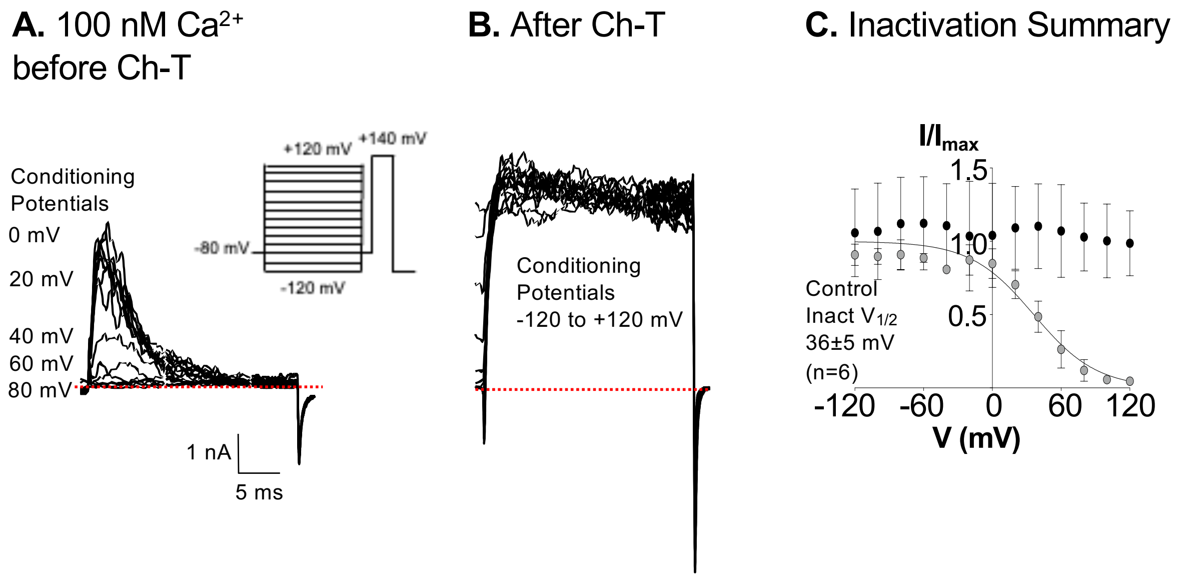

Figure S4: Steady-state inactivation in BKα:LINGO2 was completely abolished after 2 minutes of 200 μM Chloramine-T treatment. Panel A showed the steady-state inactivation of BKα:LINGO2 in 100 nM Ca^{2+} control, carried out with conditioning potentials from -120 to +120 mV (protocol inset). 200μM Chloramine-T was applied to the cytosolic side of the patches for 2 minutes to remove the inactivation and, as panel B shows, application of the same protocol failed to induce inactivation. Panel C shows a summary of 6 similar experiments in which the normalised peak current amplitude was plotted against the conditioning potential before (grey symbols) and after removal of inactivation with 200 μM Chloramine-T. Solid line shows a Boltzmann fit to the control data which yielded an inactivation $V_{1/2}$ of 36±5 mV (n=6).

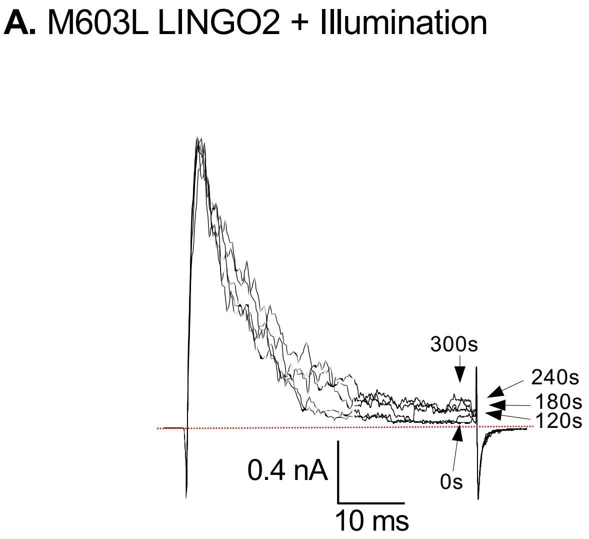

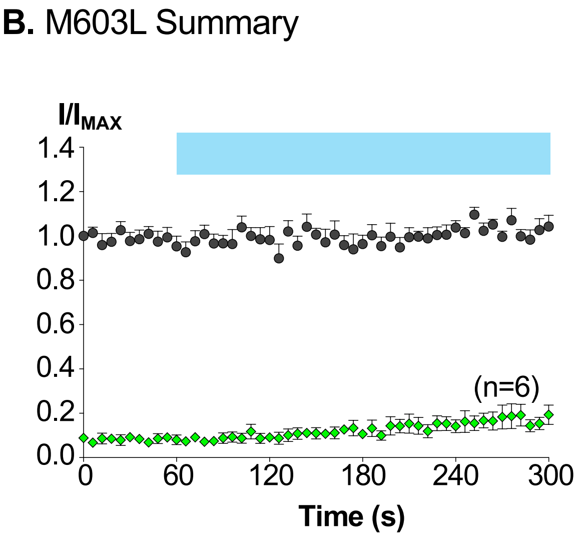

Figure S-5: Illumination of the LINGO2 M603L mutant fails to remove inactivation. Panel A shows a typical example of inactivating BK:LINGO2 M603L currents recorded in an inside out patch depolarised to +160mV for 50ms in 100 nM Ca^{2+} and then illuminated for 5 minutes. Note that there was little change in either the amplitude of the peak or sustained currents before and during illumination. Panel B shows a summary of 6 similar experiments in which the peak currents were measured in the first 5 ms (black symbols) and last 5 ms pulse (green symbols). Epifluorescent illumination was applied after 60s as denoted by the blue horizontal bar.

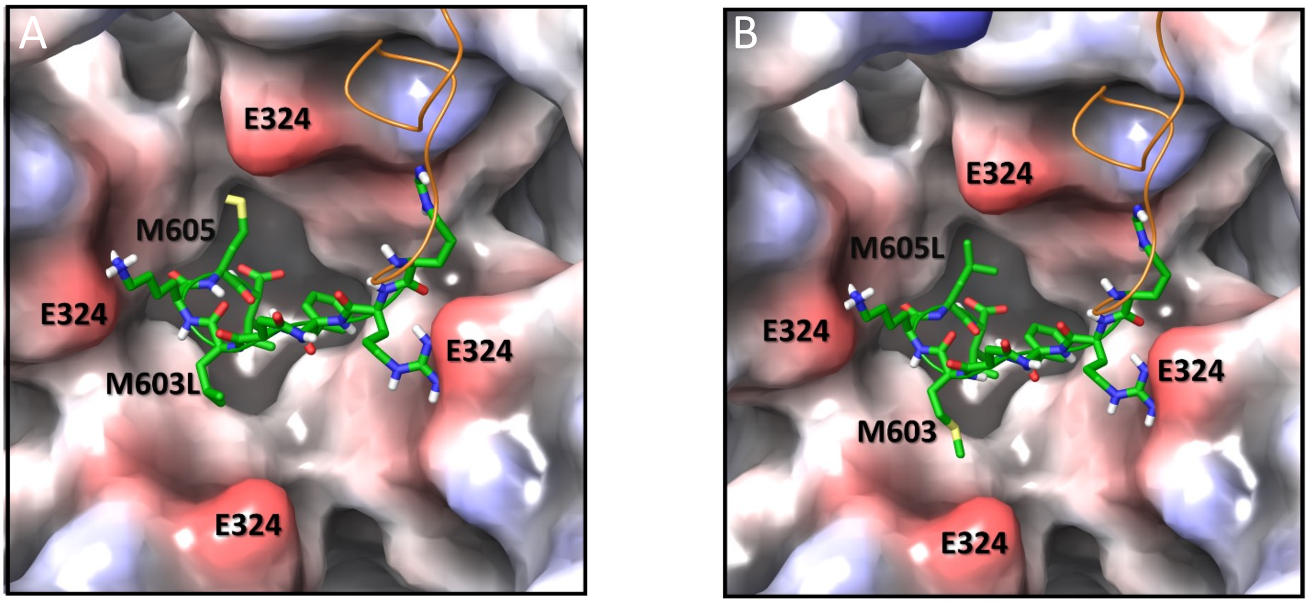

Fig S-6. The last eight residues of the LINGO2$_{M603L}$ mutant is modelled in panel A and shows that the M605 residue remains buried in a hydrophobic pocket in the pore, whereas M603L is exposed to the solvent. Panel B shows a docking for the M605L mutant in which the M603 remains solvent-exposed.
